# Verification and uniformity control of doses for ^90^Sr/^90^Y intravascular brachytherapy sources using radiochromic film dosimetry

**DOI:** 10.4103/0971-6203.41193

**Published:** 2008

**Authors:** Bayram Demir, Asm Sabbir Ahmed, Erhan Babalik, Mustafa Demir, Tevfik Gürmen

**Affiliations:** Istanbul University, Faculty of Science, Department of Physics, Istanbul, Turkey; 1 Istanbul University, Cerrahpasa Medical Faculty, Nuclear Medicine Department, Istanbul, Turkey; 2 Istanbul University, Cerrahpasa Medical Faculty, Department of Cardiology, Istanbul, Turkey; 3 Istanbul University, Institute of Cardiology, Istanbul, Turkey

**Keywords:** ^90^Sr/^90^Y, intravascular brachytherapy, radiochromic film dosimetry

## Abstract

Intravascular brachytherapy (IVBT) is a useful treatment modality for the recurrence of in-stent restenosis following drug-eluting stents (DES) or IVBT failure. The objective of this study was to measure the dose rate of ^90^Sr/^90^Y IVBT sources for comparison with that given by the manufacturer and to control the dose uniformities of these sources along the source axis. The dose rates of ^90^Sr/^90^Y beta sources were measured with a radiochromic film in a custom-made phantom. The films for calibration were irradiated using ^60^Co photon beams. The results for the three sources were 4.5%, 2.3%, and 3.5% higher than the corresponding certificate values. Maximum and minimum of the dose rates varied within ±10% of those at source center; and maximum dose discrepancy for the first ^90^Sr/^90^Y source train was 8.2%; for the second source train, 7.1%; and for the third source train, 5.1%. Our study showed that the dose rates given by the manufacturer for the three ^90^Sr/^90^Y IVBT sources were reliable and dose uniformities were within ±10% along two thirds of the treatment length.

## Introduction

Treatment modalities for in-stent restenosis include balloon angioplasty, cutting balloon angioplasty, athero-ablation techniques (excimer laser, rotational atherectomy), bare metal stent implantation, intracoronary irradiation, and drug-eluting stents (DES) implantation.[1] The two latter techniques have shown promising clinical results in recent years. Several animal experiments[2,3] and clinical studies[4,5] have indicated that the rate of recurrent restenosis can be significantly reduced to 10%-15% by irradiating the target vessel with short-range ionizing radiation at a dose range of 15-30 Gy applied locally. The technique of irradiating the vessel internally by short-range ionizing radiation is called intravascular brachytherapy (IVBT), and, in general, the isotopes of choice for IVBT are beta emitters.[6] These isotopes (e.g., ^90^Sr/^90^Y, ^32^P, ^188^W/^188^Re) are preferred because they cause insignificant irradiation to adjacent organs; and also, the personnel in the catheterization laboratory are subjected to insignificant exposure during the 3-4–minute treatment time.

Another method that prevents recurrence of restenosis is DES. DES significantly reduces in-stent restenosis rate to 5% by inhibiting neointimal hyperplasia within the stent.[7–9] But despite the high success rate of these new stents compared to IVBT,[10] failure rates up to 20% after DES implantation for the treatment of in-stent restenosis have been reported, especially in the treatment of diabetic patients.[11–13] Although there is no consensus on the mode of treatment of patients belonging to this group,[14,15] IVBT remains to be a treatment option for recurrent in-stent restenosis following DES implantation.[16–18] Additionally, recurrent restenosis is still an important challenge for up to 20% of patients previously treated with IVBT, and repeated IVBT for these patients with recurrent in-stent restenosis may be an alternative treatment modality.[19]

Clinical success of IVBT depends on the accuracy of dose delivered to the target vessel. Studies on the dosimetry of IVBT sources[20] and on the optimization of dose prescription protocols[21,23] are still continuing. Although the manufacturer provides calibration information about these sources, it is a good practice for all the departments to check all the sources, especially those having half-lives longer than 6 months.[24] For calibration purposes, the dose rate for the beta-emitting IVBT sources with a catheter-based system is normally measured at a reference point located at a radial distance of 2 mm from the source axis.[25] It is also important to know the doses along the source axis that may be necessary for the success of the treatment, especially for distal and proximal part of vessel. But manufacturer certificates do not give any information about the radiation doses at points along the source axis. ^90^Sr/^90^Y beta source trains are manufactured with lengths of 30, 40, and 60 mm; and the dose uniformities may be disrupted with the increasing of source length. American Association of Physicists in Medicine AAPM TG-60[25] recommends that the dose rate at 2-mm distance should be uniform to within ±10% over the centered two thirds of the treated length.

In the present study, we measured the dose rates of three ^90^Sr/^90^Y beta sources to compare with those indicated by their manufacturer certificates. Besides, despite the absence of dose uniformity control in the manufacturer certificate, we checked the dose uniformities at points along the source axis using radiochromic film dosimetry.

## Materials and Methods

### The ^90^Sr/^90^Y source train and irradiation system

The ^90^Sr/ ^90^Y source (Beta-Cath System, Novoste Corporation) used in IVBT to prevent restenosis is a pure beta-emitting radionuclide having emissions of average energy of 196 and 934 keV (maximum energy values are 546 and 2226 keV) respectively with a half-life of 28.5 years. The source train consists of 16 radioactive steel cylinder seeds composed of strontium titanate ceramic and two nonactive radiopaque marker seeds at each end. The seeds are 2.5×0.64 mm in size (length×diameter), and the active source length is 40 mm. When not in use, the source train is stored inside a shielding container. The diameter of delivery catheter used in the treatment is 5 French (1 French = 0.318 mm). It contains three lumens — for guide wire, radioactive seeds, and water flow. In this system, dose calculations are made according to the reference vessel diameter, and the treatment time is 3 to 4 minutes. In the calibration certificate, the company gives the dose rate at a distance of 2 mm from the source axis in water with a well-type ionization chamber. The nominal activities of the three source trains used in this study were 2.032, 2.239, and 2.640 GBq respectively.

The irradiation system mentioned above works as follows. A manual after-loading system brings the source train from the Beta-Cath unit to the distal section of the delivery catheter by hydraulic pressure. During the treatment, a cardiologist propels the radioactive seeds down the delivery catheter positioned at the treatment site using a syringe filled with sterile water. Through water pressure, the radioactive seeds are kept in this location for the duration of the treatment. At the end of the treatment time, the source train is retracted to the shielding container by reversing the water flow through the delivery catheter.

### Radiochromic film dosimetry

Standard ion chambers are not ideal for the application of IVBT dosimetry at short distances from a small source because of geometric artifacts and spatial averaging of the dose. Although well-type ionization chambers are well suited to measure the dose rate of sources of these types, radiochromic films can be used as a tool to determine the dose distributions rather than point doses. Already, some studies have shown that radiochromic film dosimetry is suitable to be performed with some type of phantoms specially designed to work with IVBT sources[26–33] and with conventional brachytherapy sources[34,35] because it is nearly tissue equivalent and has a linear optical density–dose response.

The radiochromic film is a thin, almost colorless polyester sheet embedding a chromophore that changes to dark blue under the influence of radiation. Typically, the radiochromic film is calibrated with a known radiation dose, and the relationship between dose and film response is determined. The radiochromic film used in this study was HD-810 (Nuclear Associates) and had a single layer of radiosensitive material with a thickness of 7 *µ*m on a 100-*µ*m polyester base. HD-810 films are mainly used for dose mapping in the range of 50 to 2500 Gy. For more information about radiochromic films, it is suggested that readers refer AAPM TG-55.[36]

**Figure 1:**
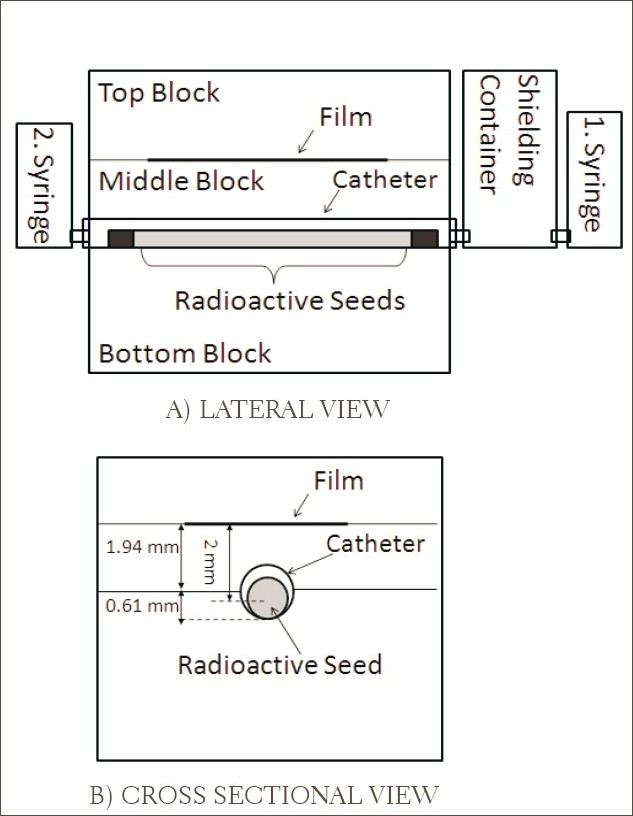
Sketch of the experimental set-up obtained for the radiochromic film measurements with a specially manufactured solid water verification block, catheter and irradiation system (not scaled). The catheter has a 0.74 mm inner diameter and 1.19 mm outer diameter.

The radiochromic film measurements were performed with a specially manufactured catheter and verification block produced by the Novoste Corporation [Figure 1]. The catheter and the verification block were designed to accurately position the source train so that the source axis was 2 mm from the radiochromic film surface. The verification block was made of solid water material (Gammex RMI 457) composed of hydrogen (8.09%), carbon (67.22%), nitrogen (2.4%), oxygen (19.84), chlorine (0.13%), and calcium (2.32%). The block had a density of 1.0467 g/cm3, and the dimensions of the block were 12×5×5.5 cm. The catheter contained one lumen for the radioactive source train.

**Figure 2:**
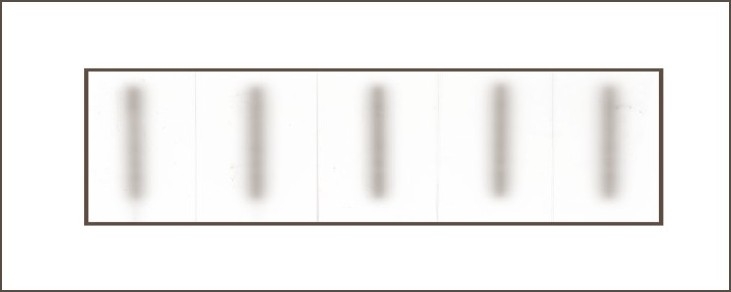
The irradiated radiochromic films obtained from five measurements with the first ^90^Sr/^90^Y beta source train during the 20-minute irradiation time

To irradiate the radiochromic film with the ^90^Sr/^90^Y source, a piece of radiochromic film (3.5×8 cm^2^) was placed inside the verification block so that its radiosensitive side faced the source. Then the source train was propelled through the catheter placed inside the phantom using a syringe filled with sterile water. (Figure 1 shows this syringe.) At the end of the 20-minute irradiation [Figure 1], the source train was retracted into the shielding container using a second sterile syringe (Figure 1 shows this second syringe) that was connected to the other end of the catheter. Irradiation times for the radiochromic films were rather higher than the treatment times due to the low sensitivity of the radiochromic film for low radiation doses. Thus the best optical densities on the films were obtained. The measurements were repeated five times for each source train under the same conditions. As examples of these films, the films irradiated with the first source are shown in Figure 2.

**Figure 3:**
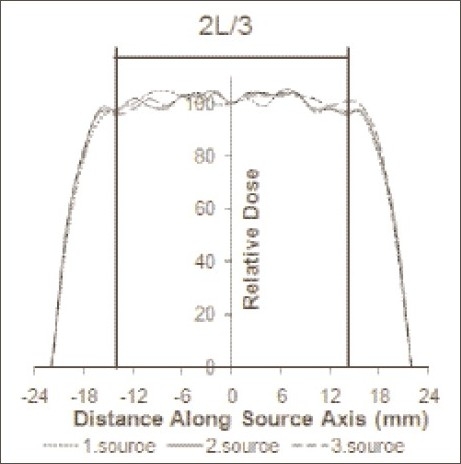
The dose profiles along the source axis at a radial distance of 2 mm (Doses are normalized to the dose values at 0 mm). (L is 40 mm and 2L/3 ≅ 27 mm)

Optical densities of films were carefully read along the source axis using a transmission densitometer. The doses at these points were calculated using calibration procedure described below. In order to make a comparison with the certificate values, the absorbed doses at the middle of the films were tabulated. The minimum, maximum, and average values of our results are presented in Table 1. Besides, the relative dose profiles were drawn as a function of distance from the center of the source [Figure 3].

**Table 1:** Results of dose rate measurements made by the radiochromic film

*Source No*	*Certificate Gy/s*	*Average of measurements Gy/s*	*Minimum measurements Gy/s*	*Maximum measurements Gy/s*
1	0.0961	0.1004 (1.045)	0.0990 (1.030)	0.1011 (1.052)
2	0.1059	0.1084 (1.023)	0.1040 (0.982)	0.1102 (1.041)
3	0.1244	0.1287 (1.035)	0.1250 (1.005)	0.1297 (1.043)

The values in parenthesis are normalized to the certi. cate value of each source

Because the radiochromic film has been shown to have response to electron beams to be the same as that to ^60^Co gamma rays (to within 3%),[34] ^60^Co beams can be used to determine the dose-film response relationship. In this study, the radiochromic films were calibrated on the same day using ^60^Co beams with 50, 100, 150, 200 Gy doses at a depth of 5 cm in a solid water phantom (20×30×30 cm^3^), which was large enough to create full-scatter environment. Optical densities of the films were measured after 24 hours, and a calibration curve was generated between the optical densities and the doses using the densitometer. The net optical densities for the films were determined by subtracting the background optical density of an unexposed film stored in the same conditions. The densitometer (model: DensiX; manufacturer: PTW-Freiburg, Germany) uses a standard fluorescent light (broadband) obtained from a simple bulb. Besides, it uses a filter for filtering the infrared light.

## Results

The results obtained for the verification of the absolute dose rate for the ^90^Sr/^90^Y source trains using the radiochromic film dosimetry are presented in Table 1. The measured values were found to be higher than those provided by the manufacturer for almost all the sources. The differences were within 10%, which is in accordance with the recommendation of AAPM TG-60.[25] The average dose rates obtained from radiochromic film dosimetry were 4.5%, 2.3%, and 3.5% higher than the certificate values for the three source trains (0.0961, 0.1059, and 0.1244 Gy/s respectively). The largest difference (+5.2%) between the certificate value and the experimental value was found for the maximum dose rate (0.1011 Gy/s) during the measurements of the first source.

We also checked the dose uniformities for three ^90^Sr/^90^Y IVBT source trains using radiochromic film dosimetry. As it was seen in Figure 3, the maximum and minimum of the dose rates for all sources differed within ±10% along two thirds of the treatment length compared to the source centers. Maximum dose discrepancy for the first ^90^Sr/^90^Y source train was 8.2%; for the second source train, 7.1%; and for the third source train, 5.1%.

### Uncertainties of results

The differences between the certificate values and our experimental results might be due to the following reasons: i) The density of the water phantom (1.0467 g/cm^3^) is 4.67% higher than that of water (1 g/cm^3^); ii) the calibration factor differs by 3% when radiochromic films are calibrated with ^60^Co beams instead of electron beams; iii) the variation of the stopping power ratio for the radiochromic film is at the most 3.5% over the considered energy ranging from 10 keV to 6 MeV, as pointed out in International Commission on Radiation Units and Measurements (ICRU) Report No. 56.[37]

## Discussion

Even though DES implantation seems to be superior to IVBT for patients with initial in-stent restenosis, recurrent restenosis is still an important challenge for patients previously treated with IVBT for the treatment of first-stent restenosis. But dosimetry of brachytherapy sources is difficult because of their physical properties and sizes. The high dose gradient of these sources makes it even more difficult to perform the dosimetric analysis. Therefore, the acceptable limits of dosimetry for brachytherapy sources are wider than those for external beam radiotherapy.[24,25]

Several studies[26–33] have been performed for the verification of the dose rates of the ^90^Sr/^90^Y source trains, produced by the Novoste Corporation, using radiochromic film dosimetry [Table 2]. One of them is the study of Duggan et al.,[26] where four ^90^Sr/^90^Y source trains (5.0 F-30 mm) and an HD-810–type radiochromic film were used. The authors reported a dose rate difference ranging from– 5.8% to +7.5% compared to the well-type ionization chamber measurements. Another is the study by Amin *et al*.,[28] where two different radiochromic films were used. They measured the dose rate of one source train (5.0 F-40 mm) using HD-810– and MD55-2–type radiochromic films. Their results were very close to the certificate values, and they reported a 1.6% higher dose rate for HD-810 film and a 4% higher dose rate for MD55-2 film as compared to the certificate. In another study, Roa et al.[31] measured the dose rate of four Novoste model ^90^Sr/^90^Y source trains (5.0 F-30 mm, 5.0 F-60 mm, 3.5 F-40 mm, and 3.5 F-30 mm) using MD55-2–type radiochromic film. They reported dose rates very close to those in the certificates (+2.8% for 5.0 F-30 mm; –0.3% for 5.0 F-60 mm; and +2.1% for 3.5 F-40 mm), except for one source (+13.7% for 3.5 F-30 mm). Most recently, Kalef-Ezra *et al*.[33] have studied the dosimetry of five ^90^Sr/^90^Y source trains (3.5 F–40-60 mm and 5.0 F–40-60 mm) using MD55-2–type radiochromic film, and they reported that the average of the measured dose rate was 8% higher than the manufacture's stated value. Although the results of these studies are close to certificate values, major differences (exceeding 10%) have also been reported in the study by Piessens and Reynaert.[27] They measured the dose rates of two ^90^Sr/^90^Y source trains (5.0 F–30-40 mm) using an HD-810–type radiochromic film and observed an average dose rate that was 20% higher than the manufacture's stated value. They had no explanation for this large discrepancy and concluded that the calibration method used by Novoste Corporation might not be accurate enough or the calibration performed by NIST (National Institute of Standards and Technology) might be systematically too low with respect to the dose rates of sources. Then NIST revised the calibration of IVBT ^90^Sr/^90^Y source trains, which resulted in an increase of the absolute dose rate by 15%. This revision brings their previously measured higher dose rates for these sources in close agreement with NIST value (within an average of +5.2%).[38] In general, the results of investigations seen in Table 2 are higher than their corresponding certificate values, but within ±10%; and our results are in general agreement with the results reported by the other investigators.

**Table 2:** Ratios (measured to certified values) of the dose rates reported by various investigators using radiochromic films for ^90^Sr/^90^Y source trains[37]

*Author*	*Number of source trains*	*Catheter type*	*Film type*	*Average of (measured/certified) of trains source Trains*
Duggan *et al*[26]	4	5.0 F	HD-810	0.996
Piessens and Reynart[27]	2	5.0 F	HD-810	1.052[*]
Amin *et al*[28]	1	5.0 F	HD-810	1.016
			MD55-2	1.040
Rosenthal *et al*[29]	3	5.0 F	HD-810	0.991
Roa *et al*[30]	2	5.0 F	MD55-2	1.012
	2	3.5 F	MD55-2	1.079
Kirisits *et al*[31]	7	5.0 F	HD-810	1.025
	6	3.5 F	HD-810	1.042
Tondeur *et al*[32]	1	5.0 F	MD55	1.080[*]
Kalef-Ezra *et al*[33]	3	5.0 F	MD55-2	1.097
	2	3.5 F	MD55-2	1.068
Present study	3	5.0 F	HD-810	1.034

* Based on the revision of dose rates given by Novoste Corporation in spring 2000.

For intravascular brachytherapy with catheter-based systems, AAPM TG-60[25] recommends that the dose rate should be uniform to within ±10% over the centered two thirds of the treated length. Duggan *et al*.[26] reported that no dose discrepancies exceeded ±10% along the source axis. They studied with four ^90^Sr/^90^Y source trains of 30 mm and checked a rectangular strip (24.5 mm long, 0.40 mm wide), on the radiochromic film irradiated at 2-mm distance from the source axis. They determined that the maximum and minimum of the dose rate along the axis changed within ±10% to that of the average dose rate. They found the dose rate discrepancies to be 10%, 8.75%, 8.3%, and 8% along the source axis at a 2-mm radial distance for four source trains respectively.

As a different study by Duggan *et al*.,[26] we checked the dose uniformities of 40-mm sources. For all source trains, we found that the dose rate uniformities were within ±10% along two thirds of the treatment length (≅27 mm). The maximum and minimum of the dose rates differed within ±10% to that at source center; and maximum dose discrepancy for the first source was 8.2%; for the second source, 7.1%; and for the third source, 5.1%.

## Conclusion

The results of our study showed that the dose rate values given by the manufacturer for the three ^90^Sr/^90^Y source trains are reliable. Although well-type ion chambers are the best choice for the dosimetric checks of IVBT source trains, radiochromic film dosimetry can also be used to verify the dose rate of the ^90^Sr/^90^Y source train with a phantom suitable for these sources in radiotherapy departments where a calibrated well-type ion chamber might not be readily available. Apart from this, we determined that the dose uniformities of these sources were within ±10% along two thirds of the treatment length (≅27 mm). However, for successful treatment with these sources, the specific dose uniformity concerning each source should be given along with the dose rate values in the manufacturer certificates and it should be checked using a suitable dosimetric tool such as radiochromic film dosimetry by a physicist.
